# Birds in My Garden

**Published:** 1915-05-15

**Authors:** 


### BIRDS IN MY GARDEN.

### By a Wounded R.A.M.C. Officer.

Peace and spring after winter and war. The contrast is pleasing, even though the enjoyment of it is hampered by a leg in splints. Though sorry to be forced from stirring scenes and the comradeship of field service, I am glad to see the buds breaking, the birds building, and the thousand-and-one events of the time of year recurring with their unvarying regularity. My own domain, placed high at the edge of the downs, is a paradise of birds. The chorus of song does not cease from dawn till after sunset, and the variety of bird life is sufficiently great to be interesting, although the want of a stream or sheet of water debars me from entertaining in my grounds many water-loving kinds that dwell near me in surroundings suited to their habits. I should much like to know how many of my bird friends are old friends. I incline to think that our commoner birds do not stray far from their selected haunt. The partial migration of common birds not classed as migrants is an established fact, but I think that the wanderers probably are chiefly the young birds that have not yet established themselves and settled down. The flocks of finches, for instance, that in winter are seen in the hedges along any country road may be the offspring of older birds that have a tenure of their nesting haunts, and remain in them year in and year out. When the nesting season comes the younger birds, by force of beak and claw, will take and hold a nesting place, but perhaps seldom can dispossess their elders. To hold property individually, despite theories of Communists, appears to be a law of Nature, and the right is recognised to an extent that emboldens the holder to oppose and drive off even a stronger trespasser and the consciousness of wrong daunts the would-be thief. Thus any blackbird or thrush will attack boldly, and usually drive off, a trespasser on his domain. It is not possible that the trespasser is always physically the feebler. It is, therefore, probable that the recognition that he is in the wrong, the want of the moral force inspired by the consciousness of right being on his side, turns the scale against him in the fight. The success with which a smaller dog will turn out a stronger dog from his master's grounds or his own kennel is another instance of the same sort.

### Birds that are Constant.

I believe many birds remain in or near my small holding throughout the year, because every spring I find many nests in exactly the same places, and in a certain number of instances peculiarities have made it possible to identify certain birds. For instance, for three springs following there has been a blackbird's nest on a certain wall in which the eggs are blue with no markings, or very slight ones, and slightly irregular in shape, being more rounded than common, and not absolutely symmetrical. The bird has no noticeable peculiarity, and the eggs are fertile. It is outside probability that more than one blackbird exhibiting this accidental and not common variation should have chanced to choose this particular ivy-clad wall for its nest. It is, therefore, all but certain that for three years at least the hen bird—for the variation of the eggs from normal shape and colouring must be due to her—has lived near and held nesting rights over this spot. There is a cock blackbird, quite half of the plumage of which is white, that has lived all the year round, for at least three years, on a road I have occasion to use. I see him frequently as I go by his haunt, and always within two or three hundred yards of the same spot. Since it is not likely that he is a bachelor, and since he is at this place in spring as well as at other seasons, it may be considered that he has established a right to nest here. Birds are seldom tolerant of a pair of the same species close to their nesting place, unless they belong to definitely social species such as rooks, but will allow birds of other species to nest in the same bush. I have had a thrush and a greenfinch nesting in one hawthorn in my garden, and a wren and a blackbird in another. Last year a hedge-sparrow and a blackbird were sitting within three feet of one another in a quickset hedge. Both hatched successfully.

### Birds that Build in Same Spot.

Last year a pair of robins built a nest in some ivy opposite my dining-room window. The nest was on the ground. The birds successfully hatched, but unfortunately a spaniel one day found the young birds and brought them to me. They were still alive, poor things, but their tender bodies were too tender to stand the pressure of even a soft-mouthed dog, and they were too damaged to be saved. This year I see the robins again busy building in the same spot. I think it must be the same pair, or one of last year's pair with a new mate, undaunted by misfortune, returned to the favoured spot. It might be argued that the birds that met misfortune to their hatch would not be so foolish as to return to it, and that therefore my contention that my friends stay constantly with me is not supported by this instance. Unfortunately, birds do not seem to learn from misfortunes.

There is a missel thrush in my orchard with a mania for building low down. The first year she built low in an apple tree. A horse rubbed the nest out or mistook it for a wisp of hay. The next year she tried a Burgundy pear, and a similar mischance befell. This year she is in the same place in the same pear tree, not five feet from the ground, and is sitting on four eggs. I wish her luck, but fear her fate. Each time after the destruction of the first nest a second was made, and the eggs hatched, in a tall fir, yet again this year she must needs tempt fortune. The limits of space forbid me to write of the blackbirds of the cattle-shed, the robins of the haystack, and other things that confirm my belief that many of my bird friends are old friends.

